# Salidroside contributes to reducing blood pressure and alleviating cerebrovascular contractile activity in diabetic Goto-Kakizaki Rats by inhibition of L-type calcium channel in smooth muscle cells

**DOI:** 10.1186/s40360-017-0135-8

**Published:** 2017-04-26

**Authors:** Yu-Guang Ma, Jun-Wei Wang, Yun-Gang Bai, Mei Liu, Man-Jiang Xie, Zhi-Jun Dai

**Affiliations:** 1 Department of Oncology, The Second Affiliated Hospital of Medical College, Xi’an Jiaotong University, Xi’an, Shaanxi Province 710004 China; 2 Department of Cardiovascular Medicine, Shaanxi Provincial People’s Hospital, Xi’an, Shaanxi 710068 China; 3 Department of Aerospace Physiology, Key Laboratory of Aerospace Medicine of Ministry of Education, Fourth Military Medical University, Xi’an, 710032 Shaanxi Province China

**Keywords:** Salidroside, Contractile activity, Vascular smooth muscle cells (VSMCs), L-type Ca^2+^ channel (Ca_L_)

## Abstract

**Background:**

Vascular disease is a common and often severe complication in diabetes mellitus. Hyperglycemia and hypertension are considered to be two of the leading risk factors for vascular complications in diabetic patients. However, few pharmacologic agents could provide a combinational therapy for controlling hyperglycemia and blood pressure in diabetic patients at the same time. Salidroside (SAL) is the major active ingredient derived from Rhodiola. Recently, it has been reported that SAL have an obvious hypoglycemic effect in diabetes and show a beneficial activity in diabetic vascular dysfunction. However, it remains unknown whether or not SAL treatment could directly reduce blood pressure in diabetes. Furthermore, it is not clear what is the molecular mechanism underlying the vascular protection of SAL treatment in diabetes.

**Methods:**

Male diabetic Goto-Kakizaki (GK) and non-diabetic control Wistar-Kyoto (WKY) rats were administrated with different dosages of SAL (50, 100 and 200 mg/kg/day) for 4 weeks. Contractile responsiveness of cerebral artery to KCl or 5-HT was investigated by Pressure Myograph System. The activity of Ca_L_ channel was investigated by recording whole-cell currents, assessing the expressions of Ca_L_ channel α_1C_-subunit and its downstream kinase, MLCK, at protein or mRNA levels.

**Results:**

We showed that administration of 100 mg/kg/day SAL for 4 weeks not only lowered blood glucose, but also reduced blood pressure and alleviated cerebrovascular contractile activity in diabetic GK rats, which suggested that SAL treatment may provide a combinational therapy for lowering blood glucose and reducing blood pressure in diabetes at the same time. Furthermore, SAL treatment markedly inhibited the function and expression of Ca_L_ channel in cerebral VSMCs isolated from diabetic GK rats or when exposed to hyperglycemia condition, which may be the underlying mechanism responsible for the vascular protection of SAL in diabetes.

**Conclusions:**

The present study provided evidences that SAL contributes to reducing blood pressure and alleviating cerebrovascular contractile activity in diabetic GK rats by inhibition of Ca_L_ channel in smooth muscle cells, which may provide a novel approach to treat vascular complications in diabetic patients.

**Electronic supplementary material:**

The online version of this article (doi:10.1186/s40360-017-0135-8) contains supplementary material, which is available to authorized users.

## Background

Vascular disease is a common and often severe complication in diabetes mellitus (DM) for it significantly increases the risk for heart attack, stroke, retinopathy, nephropathy, neuropathy, and diabetic foot disorders. Diabetic vascular complication is usually characterized by the increased vascular resistance, augmented contractile activity, impaired endothelium-dependent vasodilatation, increased oxidative stress, enhanced inflammation, and progressive atherosclerosis [1, 2]. The clustering of risk factors could accelerate the progression of diabetic vascular complication, such as hyperglycemia, high blood pressure, dyslipidaemia, insulin resistance, smoking, and obesity. There is evidence from large randomized-controlled trials that good glycemic control in both type 1 and 2 DM can delay the onset and progression of diabetic vascular complication [3, 4]. In addition, it has also been shown that the aggressive blood pressure control could significantly reduce the risk for stroke, heart failure, retinopathy, and nephropathy in DM [5–7]. At present, most diabetic patients with elevated blood pressure will require two or more agents to decrease hyperglycemia and lower blood pressure. However, few pharmacologic agents could provide a combinational therapy for controlling hyperglycemia and blood pressure in diabetic patients at the same time.

As a well-known traditional herb medicine, Rhodiola rosea (Rhodiola) has been demonstrated to have beneficial effects for high altitude-related symptoms [8]. Recently, it has been reported that Salidroside (SAL), the major active ingredient derived from Rhodiola, has multiple pharmacological activities, such as attenuating hypoxia [9], alleviating chronic hypoxic-induced pulmonary arterial hypertension [10], anti-oxidation [11, 12], anti-inflammation [13–15], anti-cancer, enhancing immune effects, and neuro-/hepato-/cardiovascular protection [16–18]. It is notable that several experimental and clinical studies have provided evidences that SAL was found to dramatically reduce blood glucose and serum insulin levels [9, 12], ameliorate insulin resistance [9], alleviate diabetic albuminuria [19], and stimulate glucose uptake in skeletal muscle cells [20]. Extensive studies suggested that SAL also showed the beneficial protection on vasodilatation in Goto-Kakizaki (GK) diabetic rats [21]. All these findings suggested that the potential efficacy of SAL might be additionally used for the treatment of diabetic vascular complication. However, it remains unknown whether or not SAL treatment could directly reduce blood pressure in diabetes. Furthermore, it is not clear what is the molecular mechanism underlying the vascular protection of SAL treatment in diabetes.

Vascular contractile activity, an important determinant of blood pressure, is mainly regulated by both Ca^2+^-independent and Ca^2+^-dependent mechanisms in vascular smooth muscle cells (VSMCs). The calcium-independent pathway of vascular contraction is mediated by the GTPase RhoA and Rho kinase. Particularly, increases in intracellular Ca^2+^ from receptor- or ion channel-activated pathways are the primary triggers to activate myosin light chain kinase (MLCK), which phosphorylates myosin light chains (MLC) and then activating myosin ATPase and leading to vascular contraction. It is considered that arterial contractility and vascular tone are predominantly controlled by membrane potential and Ca^2+^ influx via long-lasting voltage-dependent Ca^2+^ (l-type, Ca_L_) of VSMCs [1, 22]. Evidences from human and animal studies indicated that diabetic vascular complication with elevated blood pressure is tightly coupled to the impaired Ca_L_ channels in VSMCs [1, 23]. Therefore, Ca_L_ channel and its downstream molecules could be the important therapeutic targets in diabetic vascular complications [1].

This study was 1) to investigate the effects of SAL treatment on blood pressure and cerebrovascular contractile activity independent of a functional endothelium in diabetic Goto-Kakizaki (GK) rats; 2) to investigate the effects of SAL on Ca_L_ channels of cerebral VSMCs in diabetic GK rats or when exposed to hyperglycemia condition by recording whole-cell Ca_L_ currents, assessing the expressions of Ca_L_ channel α_1C_-subunit and one of its downstream kinases, MLCK, at protein or mRNA levels. Taken together, this study provided evidences for the first time that SAL contributes to reducing blood pressure and alleviating cerebrovascular contractile activity in diabetic GK rats by inhibition of Ca_L_ channel in smooth muscle cells, which may provide a novel approach to treat vascular complications in diabetic patients.

## Methods

### Animal model

Male diabetic Goto-Kakizaki (GK) rats (aged 18 weeks, *n* = 70) and non-diabetic control Wistar-Kyoto (WKY) rats (aged 18 weeks, *n* = 70) were purchased from the shanghai laboratory animal center of Chinese Academy of Science (Shanghai, China).

Goto-Kakizaki rats are a Wistar substrain and that Wistar-Kyoto rats are a reasonably matched control strain. After an adaptive feeding for 2 week, rats (20 weeks of age) were divided into 4 groups: control WKY rats (WKY), WKY rats administered with SAL (WKY + SAL), diabetic GK rats, and diabetic GK rats administered with SAL (GK + SAL). *Experiment I, Experiment II, and Experiment III* were designed to investigate the different dosages of SAL (50, 100 and 200 mg/kg/day) on blood glucose, blood pressure, and contractile activity of cerebral artery in diabetic GK rats. SAL was dissolved in DMSO to a stock concentration and then stored at −20 °C. SAL was intragasriclly administered daily for 4 weeks. The control groups were administrated with equal volume of vehicles. The dosage of SAL was based on the previous reports [12]. Fasting blood glucose was measured by glucose oxidase/peroxidase method and blood pressure was measured by the tail-cuff method [24]. Four weeks after SAL treatment, animals were anesthetized and then killed by exsanguination. All groups were caged individually in a room temperature of 23 °C.

### Examination of contractile activity

As previously described [25], the middle cerebral artery was obtained and the endothelial layer was removed by the injection of air bubbles. The artery was cannulated in a chamber [26] and then transferred to the Pressure Myograph System P110 (DMT, Denmark). After equilibration of 50 mmHg for 1 h at 37 °C, the contractile response to cumulative superfusion of KCl or 5-HT was determined as the percentage of luminal diameter relative to the baseline internal diameter.

### Isolation of VSMCs

Isolated VSMCs were obtained as described previously [27]. Briefly, the cerebral arteries including superior, middle, and basilar arteries were digested for 18 min at 37 °C with 4 mg/ml papain (Biochrom, Berlin, Germany), 2 mg/ml dithioerythritol (Amresco, St. Louis, Missouri, USA), 1 mg/ml bovien serum albumin (BSA), and 5 mM taurine. Isolated VSMCs were maintained in Ca^2+^-free PSS at 4 °C for use within 8 h.

### Electrophysiological measurements

Whole-cell Ca_L_ channel currents were measured with the conventional voltage clamp configuration [28]. There were two kinds of external solutions, i.e., *solution A* and *B. Solution A* contained (in mM) 130 NaCl, 5.4 KCl, 1 MgCl_2_, 10 BaCl_2_, 10 HEPES, and 10 glucose, equilibrated with 95% O_2_ and 5% CO_2_ at pH 7.4 adjusted with NaOH. After a seal of 2 GΩ was obtained, *solution B* was changed which contained (in mM) 75 Tris-Cl, 50 BaCl_2_, 10 HEPES, and 10 glucose, equilibrated with 95% O_2_ and 5% CO_2_ at pH 7.4 titrated with Tris base. The pipette solution contained 150 CsCl, 1 MgCl_2_, 10 EGTA, 5 HEPES, 5 Na_2_ATP, and 5 Na_2_ creatine phosphate, equilibrated with 95% O_2_ and 5% CO_2_ at pH 7.2 titrated with CsOH. All measurements were carried out at room temperature (22–24 °C).

### Evaluation the protein expression of Ca_L_ channel by Western blotting

According to our previous report [28], the protein sample of cerebral artery was run for SDS-PAGE for 80 min at 30 mA with a 8% Tris-Glycine gel (Invitrogen, Carlsbad, California, USA). After separation, proteins were transferred onto a nitrocellulose membrane and then blocked overnight at 4 °C. The membranes were incubated with rabbit polyclonal antibody against the Ca_L_ channel α_1C_-subunit (Alomone Labs, Jerusalem, Israel) and subsequently incubated with Infrared (IR)-labeled secondary antibodies (LI-COR). A monoclonal mouse antibody raised against β-actin (Sigma) was used as a lane-loading control. The bound antibody was detected by the Odyssey infrared imaging system (LI-COR). Densitometry analysis of bands was performed by Scion image (Scion, Frederick, MD).

### Cell culture

As previously described [29], isolated VSMCs were cultured in DMEM (GIBCO, USA) supplemented with 20% FBS (HyClone, USA). To diminish the interferences of serum, the VSMCs were cultured in 5% FBS + 3% Insulin-Transferrin-Selenium (ITS) during the pharmacological experiments. The cells at passages 5–8 were used for experimentations.

### Evaluation the mRNA expression of Ca_L_ and MLCK by Real-Time PCR

As described previously [30], total RNA was extracted from cerebral arteries or cultured VSMCs with TRIzol reagent (Invitrogen) according to manufacturer’s protocol. The concentration of RNA were determined by assessing the absorbance at 260 and 280 nm. Then RNA was reverse-transcribed into cDNA using Primerscript RT Kit (TaKaRa Biotechnology, Dalian, China). Real-time quantitative PCR analysis was performed on an ABI 7500 real-time PCR system (Applied Biosystems) using SYBR®Premix Ex TaqII Kit (Takara, Japan). The endogenous β-actin was used to evaluate the efficiency of reverse transcription. Cycle conditions were as follows: 95 °C for 30 s followed by 40 cycles (95 °C denaturation for 5 s, 60 °C annealing for 30 s). A relative quantification method (2^-△△CT^, where Ct is cycle threshold) was chosen for quantitative analysis. The relative quantitation value of target, normalized to the endogenous control β-actin. The primers pairs of Ca_V_1.2: Forward-5′-TGC TGT GTC TGA CCC TGA AG-3′ and Reverse-5′-CGT CTT CCG GAA AGG GAA TA-3′. The primers pairs of MLCK: Forward-5′-GAC GTG TTC ACC CTG GTT CT-3′ and Reverse-5′-TTT GTG CAG CAT CAG TGA CA-3′. The primers pairs of β-actin were Forward-5′-TCA GGT CAT CAC TAT CGG CAAT-3′ and Reverse-5′-AAA GAA AGG GTG TAA AAC GCA-3′.

### Statistical analysis

Followed by a S-N-K-*Post Hoc*, the differences of Ca_L_ channel current densities were determined by one-way ANOVA in different groups. The differences of body weights, blood glucose, the resting Ca^2+^ fluorescence intensity, and the maximal increase of Ca^2+^ fluorescence intensity were determined by Student’s *t*-test in different groups. A value of *P* ≤ 0.05 was used for the statistically significant.

## Results

### Physical characteristics of experimental animals

The GK rat is a spontaneous diabetic animal model with elevated blood glucose, peripheral insulin resistance, and a non-obese phenotype. In the present study, the levels of blood glucose significantly increased in diabetic GK rats, whereas there was no significant different in the body weights between age matched control WKY and diabetic GK rats, which were in consistence with previous report [21, 31, 32]. In *Experiment I*, 50 mg/kg/day SAL for 4 weeks had no obvious effects on blood glucose in WKY or GK rats, respectively, which indicated that 50 mg/kg/day SAL is not an effective dosage for reducing blood glucose in diabetic GK rats. In *Experiment II*, 100 mg/kg/day SAL for 4 weeks markedly decreased the blood glucose of diabetic GK rats and had no obvious effects in control WKY rats, which is well in accordance with earlier reports [9, 15, 19]. However, there was also a significant difference in the blood glucose between GK + SAL and WKY rats, which indicated that SAL treatment did not restore the blood glucose to the normal control level. In *Experiment III*, chronic administration of 200 mg/kg/day SAL for 4 weeks significantly decreased blood glucose in both WKY and GK rats, respectively (Table 1). In addition, there were no significant differences in the body weights among WKY, WKY + SAL, GK, and GK + SAL rats when the rats were treated with different dosages of 50, 100, and 200 mg/kg/day SAL for 4 weeks. These results indicated that dosage of 100 mg/kg/day SAL is appropriate and has an effective hypoglycemic activity in diabetic GK rats.

**Table 1:** Body weight and fasting blood glucose in WKY, WKY + SAL, GK, and GK + SAL rats in *Experiment I, Experiment II, and Experiment III*

	20-week of age		24-week of age (4 weeks SAL treatment)	
	Body weight (g)	Blood glucose (mM)	Body weight (g)	Blood glucose (mM)
*Experiment I*				
WKY(*n* = 10)	375.0 ± 11.5	5.2 ± 1.7	418.0 ± 18.8	5.2 ± 1.3
WKY + 50 mg/kg/day SAL(*n* = 10)	383.8 ± 10.2	5.4 ± 1.3	409.5 ± 15.5	4.9 ± 1.8
GK(*n* = 10)	389.0 ± 11.6	9.2 ± 2.4*	427.0 ± 12.3	11.6 ± 1.9*
GK + 50 mg/kg/day SAL(*n* = 10)	391.0 ± 11.4	8.6 ± 2.1*	419.0 ± 18.9	10.2 ± 1.3*
*Experiment II*				
WKY(*n* = 15)	386.0 ± 15	5.26 ± 1.2	412.5 ± 17.8	5.8 ± 0.9
WKY + 100 mg/kg/day SAL(*n* = 15)	378.5 ± 13	5.4 ± 2.2	426.9 ± 15.3	5.7 ± 1.2
GK(*n* = 15)	392.0 ± 18	8.9 ± 1.3*	431.3 ± 16.3	10.5 ± 1.4*
GK + 100 mg/kg/day SAL(*n* = 15)	387.0 ± 17	9.1 ± 1.6*	425.0 ± 11.2	7.8 ± 1.5^#^*
*Experiment III*				
WKY(*n* = 10)	378.0 ± 11.0	4.7 ± 1.5	408.0 ± 17.2	5.8 ± 1.6
WKY + 200 mg/kg/day SAL(*n* = 10)	383.2 ± 13.6	5.2 ± 1.1	415.5 ± 16.5	3.4 ± 1.8*
GK(*n* = 10)	389.0 ± 12.7	8.5 ± 1.2*	436.0 ± 12.7	11.2 ± 1.5*
GK + 200 mg/kg/day SAL(*n* = 10)	393.0 ± 16.5	8.9 ± 1.0*	428.0 ± 15.2	8.1 ± 3.3^#*^

*WKY* Control WKY rats, *WKY + SAL* WKY rats administrated with SAL, *GK* Diabetic GK rats, *GK + SAL* GK rats administrated with SAL. SAL was administrated with different dosage of 50, 100 and 200 mg/kg/day for 4 weeks in *Experiment I, Experiment II, and Experiment III*, respectively. *P < 0.05 vs. WKY and ^#^ *P <* 0.05 vs. GK rats

### Chronic administration of 100 mg/kg/day SAL for 4 weeks significantly reduced systolic and diastolic blood pressure in diabetic GK rats

**Fig. 1:**
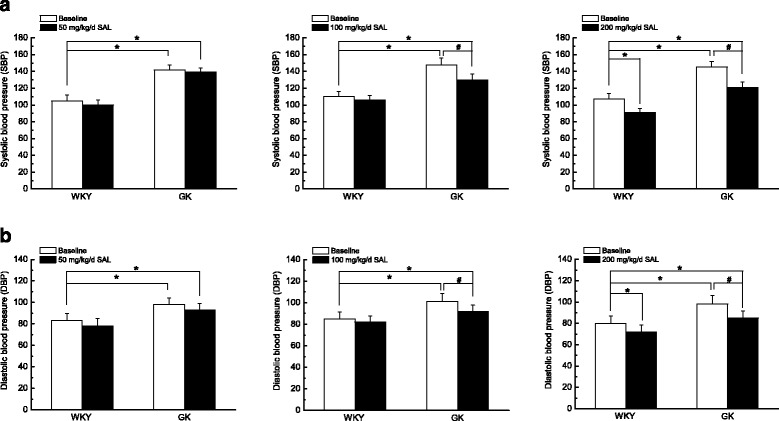
Comparison of systolic (**a**) and diastolic blood pressure (**b**) from WKY, WKY + SAL, GK, and GK + SAL rats. SAL was administrated with different dosage of 50, 100 and 200 mg/kg/day for 4 weeks in *Experiment I, Experiment II, and Experiment III*, respectively. Chronic administration of 50 mg/kg/day SAL had no obvious effects on systolic (**a**) and diastolic blood pressure (**b**) in WKY or GK rats, respectively. However, chronic administration of 100 mg/kg/day SAL significantly reduced the blood pressure in diabetic GK, whereas did not affect the blood pressure in control WKY rats. In addition, chronic administration of 200 mg/kg/day SAL significantly decreased systolic (**a**) and diastolic blood pressure (**b**) in both WKY and GK rats, respectively. These results indicated that dosage of 100 mg/kg/day SAL is effective for lowering blood pressure in diabetic GK rats. Values are expressed as means ± SEM and *n* = 10 animals in each group. *P < 0.05 vs. WKY rats and #*P <* 0.05 vs. GK rats

As compared with control WKY rats, diabetic GK rats showed a significant increase in systolic (Fig. 1a) and diastolic blood pressure (Fig. 1b), which is consistence with previous report [21]. In *Experiment I*, chronic administration of 50 mg/kg/day SAL for 4 weeks did not affect the blood pressure in WKY or GK rats, respectively. In *Experiment II*, chronic administration of 100 mg/kg/day SAL for 4 weeks significantly reduced the blood pressure in diabetic GK, whereas did not affect the blood pressure in control WKY rats. In *Experiment III*, chronic administration of 200 mg/kg/day SAL for 4 weeks significantly decreased blood pressure in both WKY and GK rats, respectively. These results indicated that dosage of 100 mg/kg/day SAL is effective for reducing blood pressure in diabetic GK rats.

### Chronic administration of 100 mg/kg/day SAL for 4 weeks markedly inhibited the contractile activity of middle cerebral artery in diabetic GK rats

**Fig. 2:**
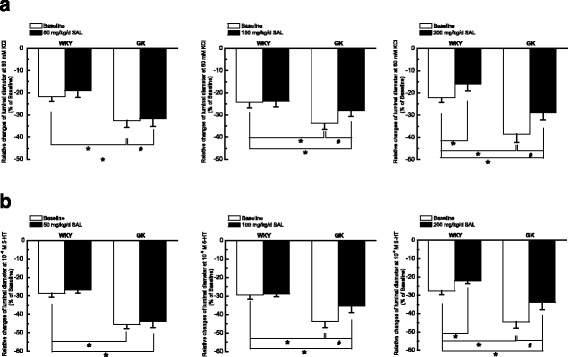
Comparison of contractile function in response to 60 mM KCl (**a**) and 10^−6^ M 5-hydroxytryptamine (5-HT, **b**) in middle cerebral artery isolated from WKY, WKY + SAL, GK, and GK + SAL rats, respectively. SAL was administrated with different dosage of 50, 100 and 200 mg/kg/day for 4 weeks in *Experiment I, Experiment II, and Experiment III*, respectively. Chronic administration of 50 mg/kg/day SAL had no obvious effects on contractile responsiveness to KCl (A) and 5-HT (**b**) in WKY or GK rats, respectively. However, chronic administration of 100 mg/kg/day SAL significantly inhibited the augmented contractile responsiveness to KCl (**a**) and 5-HT (**b**) in GK rats, whereas did not change the contractile responsiveness in WKY rats. In addition, chronic administration of 200 mg/kg/day SAL significantly inhibited the contractile responsiveness to KCl (**a**) and 5-HT (**b**) in both WKY and GK rats, respectively. WKY: control WKY rats, WKY + SAL: control WKY rats administrated with SAL, GK: diabetic GK rats, GK + SAL: GK rats administrated with SAL. Values are expressed as means ± SEM and *n* = 8 animals in each group. *P < 0.05 vs. WKY rats and #*P <* 0.05 vs. GK rats

**Fig. 3:**
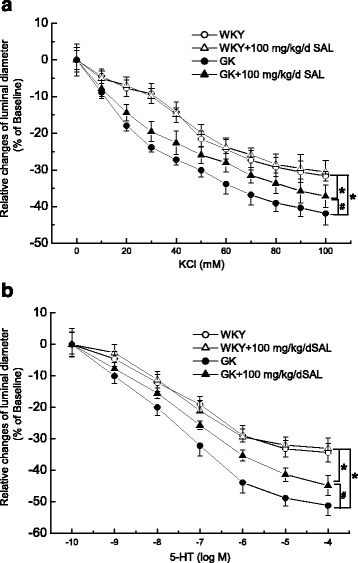
Chronic administration of 100 mg/kg/day SAL significantly inhibited the contractile responsiveness of middle cerebral artery to cumulative superfusion of KCl (**a**) and 5-hydroxytryptamine (5-HT, **b**) in diabetic GK rats. Concentration-response relationships of middle cerebral artery were represented as the percentage of luminal diameter relative to the baseline internal diameter. WKY: control WKY rats, WKY + SAL: control WKY rats administrated with 100 mg/kg/day SAL, GK: diabetic GK rats, GK + SAL: GK rats administrated with 100 mg/kg/day SAL. Values are expressed as means ± SEM and *n* = 8 animals in each group. *P < 0.05 vs. WKY rats and #*P <* 0.05 vs. GK rats

The endothelial layer was removed to rule out the endothelium-dependent relaxation. As compared with that in WKY rats, contractile responsiveness of middle cerebral artery to KCl (Figs. 2a and 3a) and 5-HT (Figs. 2b and 3b) both significantly increased in diabetic GK rats, which is in accordance with previous report [33]. In *Experiment I*, chronic treatment with 50 mg/kg/day SAL for 4 weeks had no obvious effects on contractile activity in WKY or Diabetic GK rats, respectively (Fig. 2), which indicated that 50 mg/kg/day SAL is not an effective dosage for reducing contractile activity in diabetic GK rats. In *Experiment II*, chronic administration of 100 mg/kg/day SAL for 4 weeks significantly decreased the contractile activity of middle cerebral artery to Ach (Figs. 2a and 3a) and SNP (Figs. 2b and 3b) in diabetic GK rats, respectively, whereas did not affect the contractile activity in control WKY rats. Under the condition of 60 mM KCl, chronic administration of 100 mg/kg/day SAL markedly reduced the luminal diameter from (−33.8 ± 2.6)% in GK rats to (−28.0 ± 2.1)% in GK + SAL rats (Fig. 2a). In addition, when the concentration of 5-HT was 10^−6^ M, chronic administration of 100 mg/kg/day SAL significantly decreased the luminal diameter from (−43.8 ± 2.7)% in GK rats to (−35.3 ± 3.6)% in GK + SAL rats (Fig. 2b). However, SAL treatment did not restore the contractile responsiveness in diabetic rats to the normal control level for there was also a significant difference in the contractile responsiveness of middle cerebral artery between GK + SAL and WKY rats. When the control rats were treated with 100 mg/kg/day SAL, there were no significant differences in the contractile responsiveness of middle cerebral artery between WKY + SAL and WKY rats (Fig. 3). In *Experiment III,* chronic treatment with 200 mg/kg/day SAL for 4 weeks significantly decreased the contractile responsiveness in both WKY and Diabetic GK rats, respectively (Fig. 2). These results indicated that dosage of 100 mg/kg/day SAL is appropriate for reducing contractile activity of middle cerebral artery in diabetic GK rats. Therefore, we selected 100 mg/kg/day of SAL as the working dosage in the next animal study.

### Chronic administration of 100 mg/kg/day SAL for 4 weeks markedly decreased the Ca_L_ current densities of cerebral VSMCs isolated from diabetic GK rats

**Fig. 4:**
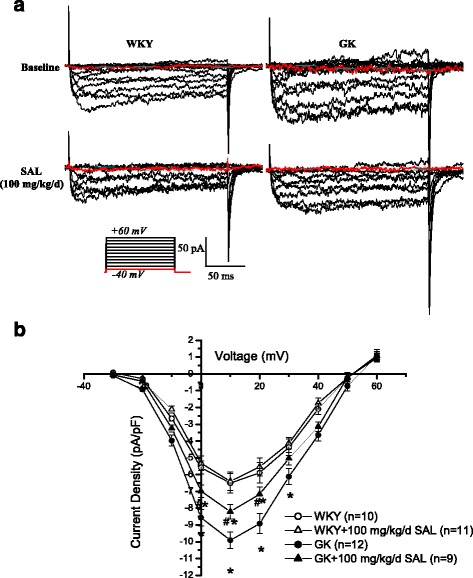
Chronic administration of 100 mg/kg/day SAL markedly decreased whole-cell Ca_L_ current densities of cerebral VSMCs isolated from diabetic GK rats. Representative recording traces were used to show the whole-cell Ca_L_ currents (**a**) and the mean *I-V* curves were further expressed in terms of current densities (**b**) in WKY, WKY + SAL, GK, and GK + SAL rats. WKY: control WKY rats, WKY + SAL: control WKY rats administrated with 100 mg/kg/day SAL, GK: diabetic GK rats, GK + SAL: GK rats administrated with 100 mg/kg/day SAL. Values are means ± SEM with the number of cells recorded in parentheses. *P < 0.05 vs. WKY rats and #*P <* 0.05 vs. GK rats

The inward currents were evoked by increasing depolarization from −40 mV to +60 mV (Fig. 4a). The mean current-voltage relationship (*I-V)* curves were expressed in terms of current densities which were calculated by normalizing current to Cm (Fig. 4b) [27]. As compared with that in WKY, whole-cell Ca_L_ currents of cerebral VSMCs are much bigger in diabetic GK rats, which is consistent with the previous reports [22, 33]. Chronic administration of 100 mg/kg/day SAL significantly decreased the Ca_L_ channel current densities of cerebral VSMCs isolated from diabetic GK rats. However, sAL treatment did not restore Ca_L_ channel current densities to the normal control level for there was also a significant difference in Ca_L_ channel current densities of cerebral VSMCs between GK + SAL and WKY rats. In addition, 100 mg/kg/day SAL had no effects in the Ca_L_ channel current densities of cerebral VSMCs in WKY + SAL or WKY rats, respectively. These results clearly suggested that chronic SAL treatment significantly decreased the Ca_L_ channel current densities of cerebral VSMCs isolated from diabetic GK rats.

### Chronic administration of 100 mg/kg/day SAL for 4 weeks significantly reduced the expressions of Ca_L_ α_1C_-subunit at protein and mRNA levels in cerebral arteries isolated from diabetic GK rats

**Fig. 5:**
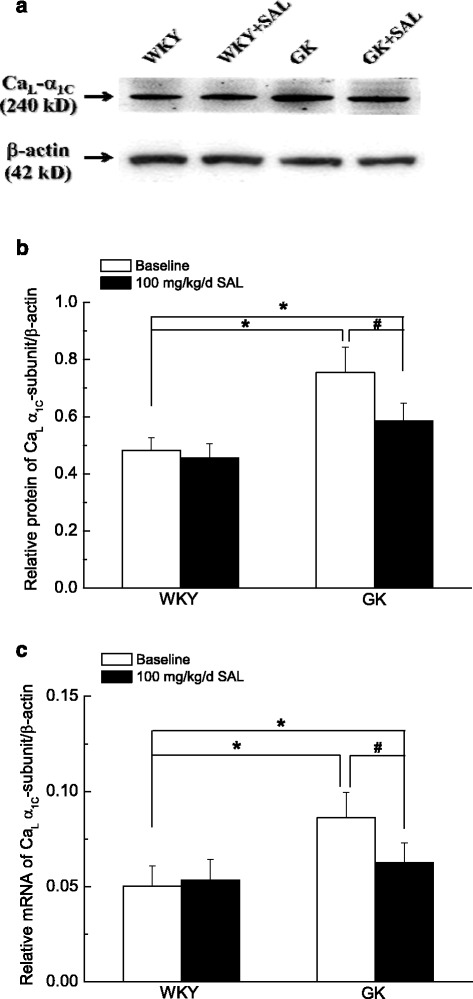
Chronic administration of 100 mg/kg/day SAL significantly reduced the expressions of Ca_L_ α_1C_-subunit at protein and mRNA levels in cerebral arteries isolated from diabetic GK rats. Representative band was used to show the protein expressions of Ca_L_ channel in WKY, WKY + SAL, GK, and GK + SAL rats (**a**). Normalized band intensities of Ca_L_ α_1C_-subunit are shown as a percentage of the β-actin density (**b**). Relative mRNA of Ca_L_ α_1C_-subunit are shown as a percentage of the β-actin mRNA (**c**). WKY: control WKY rats, WKY + SAL: control WKY rats administrated with 100 mg/kg/day SAL, GK: diabetic GK rats, GK + SAL: GK rats administrated with 100 mg/kg/day SAL. Values are expressed as means ± SEM from 4 independent experiments, and each sample based on tissue pooled from 3 to 4 animals. *P < 0.05 vs. WKY rats and #*P <* 0.05 vs. GK rats

The protein expressions of Ca_L_ α_1C_-subunit (240 kD) and β-actin (42 kDa, the internal control) were shown in the membrane in Fig. 5a [28]. The relative protein and mRNA expressions of Ca_L_ α_1C_-subunit were shown in Fig. 5b and c, respectively. As compared with that in WKY rats, expressions of Ca_L_ α_1C_-subunit significantly increased in cerebral arteries of diabetic GK rats at protein and mRNA levels, respectively. Chronic administration of 100 mg/kg/day SAL significantly reduced the α_1C_-subunit expressions in cerebral arteries of diabetic rats at both protein and mRNA levels. However, SAL treatment did not restore the α_1C_-subunit expressions of diabetic GK rat to the normal control level for there were also significant differences in α_1C_-subunit expressions of Ca_L_ channel in cerebral arteries between GK + SAL and WKY rats. When the WKY rats were treated with 100 mg/kg/day SAL, there were no significant differences in Ca_L_ α_1C_-subunit expressions between WKY + SAL and WKY rats. These results suggested that 100 mg/kg/day SAL treatment significantly reduced the α_1C_-subunit expressions of Ca_L_ channel at protein and mRNA levels in cerebral arteries isolated from diabetic GK rats.

### Acute application of SAL directly induced vasodilatation of cerebral artery isolated from normal WKY rats by inhibition of Ca_L_ channel under hyperglycemia condition

**Fig. 6:**
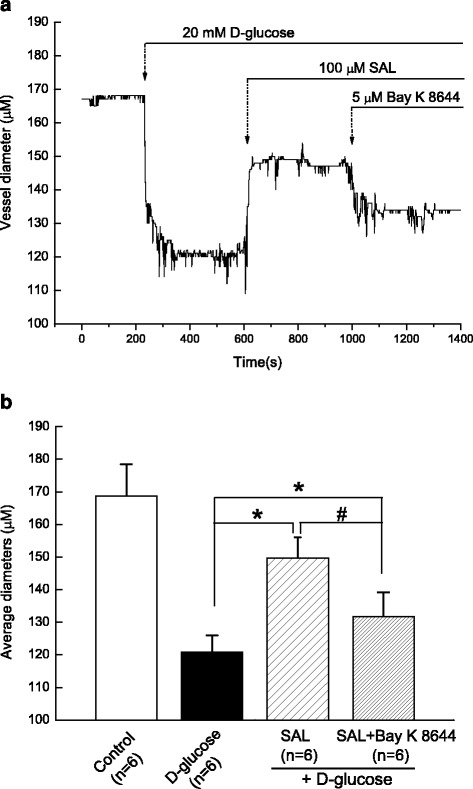
Acute application of SAL directly induced vasodilatation of middle cerebral artery isolated from normal WKY rats by inhibition of Ca_L_ channel under hyperglycemia condition. Representative diameters of middle cerebral artery were recorded before and after application of 20 mM d-glucose, 100 μM SAL, and 5 μM Bay K 8644 (the specific agonist of Ca_L_ channel), respectively (**a**). Summary data for average diameters before and after application of 20 mM d-glucose, 100 μM SAL, and 5 μM Bay K 8644 (**b**). 20 mM d-glucose induced the contraction of isolated middle cerebral artery ring and then 100 μM SAL was added as indicated dose when the contraction was stable. The SAL-induced relaxation was diminished by the addition of Bay K 8644, the specific agonist of Ca_L_ channel. Values are means ± SEM and *n* = 8 from 6 animals. *P < 0.05 vs. control condition and #*P <* 0.05 vs. high glucose condition

The middle cerebral artery was isolated from WKY rats and then the endothelial layer of middle cerebral artery was mechanically removed by the injection of air bubbles. As shown in Fig. 6, extracellular application of the hyperglycemia (d-glucose, 20 mM for 10 min [26]) obviously induced the constriction of middle cerebral artery. When the contraction reached the peak and kept in stable state, acute extracellular application of 100 μM SAL (as described previously [9, 31]) markedly induced the vascular relaxation in the presence of 20 mM d-glucose. Lastly, extracellular application of 5μM Bay K 8644, the specific agonist of Ca_L_ channel, significantly blocked the SAL-induced relaxation of middle cerebral artery under hyperglycemia condition. These findings suggested that hyperglycemia induced cerebrovascular constriction and then the acute extracellular application of SAL could directly induce vasodilatation of cerebral artery by inhibition of Ca_L_ channel under hyperglycemia condition.

### Acute application of SAL directly inhibited the Ca_L_ currents in cerebral VSMCs isolated from normal WKY rats under hyperglycemia condition

**Fig. 7:**
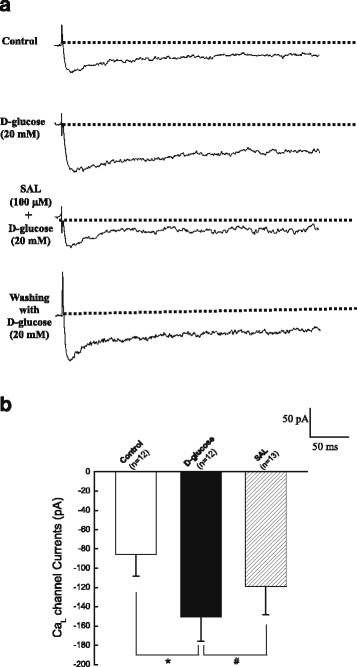
Acute application of SAL directly inhibited the Ca_L_ currents in cerebral VSMCs isolated from normal WKY rats under hyperglycemia condition. Representative traces of Ca_L_ channel current were recorded at +10 mV in cerebral VSMCs when exposed to 20 mM d-glucose and then subsequent acute application of 100 μM SAL in the presence of 20 mM d-glucose. Lastly, washing with 20 mM d-glucose could diminish the effects of SAL on Ca_L_ currents (A). Summarized data indicated the amplitudes of Ca_L_ channel current at +10 mV before and after application of 20 mM d-glucose and 100 μM SAL, respectively. Values are means ± SEM with the number of cells recorded in parentheses. *P < 0.05 vs. control condition and #*P <* 0.05 vs. high glucose condition

As shown in Fig. 7, acute application of high glucose evoked an increase in Ca_L_ channel currents at +10 mV potential. Subsequently, acute application of 100 μM SAL markedly reduced the amplitude of Ca_L_ channel currents under 20 mM d-glucose condition. Lastly, washing with 20 mM d-glucose could diminish the effects of SAL on Ca_L_ currents. These findings suggested that hyperglycemia increased the function of Ca_L_ channel and then extracellular application of SAL directly reduced the function of Ca_L_ in cerebral VSMCs under hyperglycemia condition.

### Treatment with SAL inhibited the mRNA expressions of Ca_L_ α_1C_-subunit and MLCK in cultured cerebral VSMCs under hyperglycemia condition

**Fig. 8:**
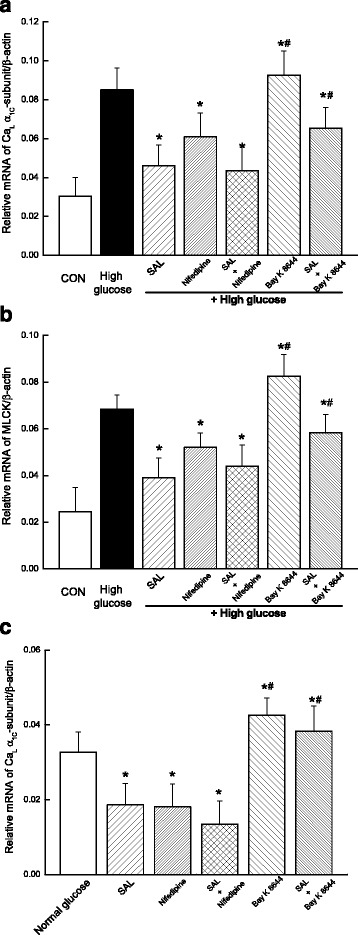
Treatment with SAL for 48 h inhibited the mRNA expressions of Ca_L_ α_1C_-subunit and MLCK in cultured cerebral VSMCs under high glucose condition. Relative mRNA levels of Ca_L_ α_1C_-subunit (**a**) and MLCK (**b**) are shown as a percentage of the β-actin mRNA, respectively. It is shown the relative mRNA levels of Ca_L_ α_1C_-subunit (**c**) in cultured cerebral VSMCs under normal glucose condition. Values are expressed as means ± SEM from 4 independent experiments. *P ≤ 0.05 as compared with high glucose control and #*P <* 0.05 vs. treatment with SAL treatment

Furthermore, we investigated the effects of SAL treatment on the expressions of Ca_L_ channel and one of its downstream kinases, MLCK, in cultured cerebral VSMCs under hyperglycemia condition. As shown in Fig. 8, there was an increased mRNA expressions of Ca_L_ α_1C_-subunit (Fig. 8a) and MLCK (Fig. 8b) in cultured cerebral VSMCs under hyperglycemia for 48 h. However, the application with 100 μM SAL for 48 h significantly decreased the mRNA expressions of Ca_L_ α_1C_-subunit and MLCK by 47 or 43% in cultured cerebral VSMCs under high glucose condition, respectively. It is interesting that blocking Ca_L_ channel with 100 nM nifedipine (the specific antagonist of Ca_L_ channel) has similar effects to the treatment with SAL, which decreased the mRNA expressions of Ca_L_ α_1C_-subunit and MLCK by 28 or 24% in cultured cerebral VSMCs under high glucose condition, respectively. Combination of SAL with nifedipine has no significant differences in the mRNA expressions of Ca_L_ α_1C_-subunit and MLCK as compared with SAL treatment alone in cultured cerebral VSMCs under high glucose condition. In contrast, application of Bay K 8644 (the specific agonist of Ca_L_ channel) significantly increased the mRNA expressions of Ca_L_ α_1C_-subunit and MLCK under high glucose condition, respectively. Combination of SAL with Bay K 8644 significantly inhibited the effects of Bay K 8644 in mRNA expressions of Ca_L_ α_1C_-subunit and MLCK under high glucose condition, respectively. Our observations indicated that treatment with SAL could directly inhibit the mRNA expressions of Ca_L_ α_1C_-subunit and MLCK in cultured cerebral VSMCs under high glucose condition.

In addition, we investigated whether salidroside or nifedipine altered the channel expression in normal glucose conditions. As shown in Fig. 8c, application with 100 μM SAL and 100 nM nifedipine for 48 h significantly decreased the mRNA expressions of Ca_L_ α1C-subunit in cultured cerebral VSMCs under normal glucose condition, respectively. Combination of SAL with nifedipine has no significant differences in the mRNA expressions of Ca_L_ α1C-subunit as compared with SAL treatment alone in cultured cerebral VSMCs under normal glucose condition. In contrast, application of Bay K 8644 (the specific agonist of CaL channel) significantly increased the mRNA expressions of Ca_L_ α1C-subunit under normal condition, respectively. Combination of SAL with Bay K 8644 significantly inhibited the effects of Bay K 8644 in mRNA expressions of Ca_L_ α1C-subunit in normal glucose condition. These results indicated that 100 μM SAL or 100 nM nifedipine induced the similar effects on mRNA expressions of Ca_L_ α1C-subunit in normal or high glucose condition.

## Discussion

There are two novel findings in the present work. First, chronic administration of 100 mg/kg/day SAL for 4 weeks not only lowered blood glucose, but also reduced blood pressure and alleviated cerebrovascular contractile activity in diabetic GK rats, which suggested that SAL treatment may provide a combinational therapy for lowering blood glucose and reducing blood pressure in diabetes at the same time. Secondly, SAL treatment markedly inhibited the function and expression of Ca_L_ channel in cerebral VSMCs isolated from diabetic GK rats or when exposed to hyperglycemia condition, which may be the underlying mechanism responsible for the vascular protection of SAL in diabetes. Taken together, the present study provided evidences that SAL contributes to reducing blood pressure and alleviating cerebrovascular contractile activity in diabetic GK rats by inhibition of Ca_L_ channel in smooth muscle cells, which might provide a novel approach to treat vascular complications in diabetic patients.

### Hyperglycemia, hypertension, and impaired Ca_L_ channel in diabetic vascular complications

Diabetes vascular complications are divided into microvascular and macrovascular complications. Microvascular complications are due to damage to small blood vessels including retinopathy, nephropathy, neuropathy, and diabetic foot disorders. Macrovascular complications are due to damage to larger blood vessels, such as heart attacks, strokes and, insufficiency in blood flow to legs [1]. Although the cellular mechanisms are complex, hyperglycemia and hypertension are considered to be two of the leading risk factors for vascular complications in diabetic patients [34]. It is well demonstrated in several randomized controlled trials and observational studies that hyperglycemia and hypertension frequently coexist, and their combination provides additive increases in the risk of life-threatening cardiovascular events in diabetes. Therefore, early recognition and treatment of both hyperglycemia and hypertension may remain central to delaying the onset and slowing the progress of diabetic vascular complication [7]. It is not clear why the diabetic subjects have an increased susceptibility to hypertension, but the mechanisms may involve impaired autoregulation and increased arterial contractility [35]. It is currently believed that increased arterial contractility and enhanced vascular tone in diabetes may arise from impaired Ca_L_ channel function [1, 22].

Ca_L_ channel is one of the important mediators to control arterial contraction by handling intracellular Ca^2+^ in VSMCs. When the membrane is depolarized, extracellular Ca^2+^ passes inward through Ca_L_ channels and then triggers calcium release by activating ryanodine receptor 2 (RyR2). The increased intracellular Ca^2+^ binds with high affinity to calmodulin (CaM), a homolog of troponin C. The calcium-calmodulin complex causes calcium-dependent protein kinases to catalyse the phosphorylation of MLCK and then subsequently leads to the phosphorylation on ser19 and thr18 of MLC, resulting in the vascular contraction by the molecular motor activity of myosin and actin. Therefore, Ca_L_ channels play a key role in regulating Ca^2+^ entry into VSMCs and thus alterations in the activity of Ca_L_ channel may strongly affect vascular contraction.

As compared with that in WKY rats, the diabetic GK rats showed a higher blood glucose level (Table 1) and an elevated blood pressure (Fig. 1) with a significant increased contractile activity of cerebral artery (Figs. 2 and 3) in the present study. In addition, the function (Fig. 4) and expressions (Fig. 5) of Ca_L_ channel significantly increased in cerebral VSMCs isolated from diabetic GK rats. Correspondingly, we also observed that hyperglycemia directly induced the cerebrovascular contraction (Fig. 6) and enhanced the function of Ca_L_ channel (Fig. 7) in cerebral VSMCs isolated from normal WKY rats. Furthermore, hyperglycemia could significantly increase the mRNA levels of Ca_L_ α_1C_-subunit and its downstream kinase, MLCK, in cultured cerebral VSMCs (Fig. 8). Our results are in agreement with previous reports that hyperglycemia impaired Ca_L_ channel, which led to the increased contractility of arterial myocytes and enhanced vascular tone during diabetes mellitus [1, 26, 36].

### The SAL treatment reduced blood pressure and alleviated cerebrovascular contractility in diabetic GK rats by inhibiting Ca_L_ channel in VSMCs

Rhodiola is a perennial plant which grows in the Arctic and high altitudes of Europe, Asia, and North America. Rhodiola has been used not only for high altitude-related symptoms, but also for improving physical and mental performance, reducing fatigue and depression, relieving vertigo and drowsiness, and alleviating chest tightness and palpitations [8]. SAL (p-hydroxyphenethyl-β-d-glucoside), a kind of phenylethanoid derivatives, is thought to be the most critical compounds extracted from Rhodiola. Recently, SAL has been demonstrated to have an obvious hypoglycemic effect in diabetes [9, 12] and a beneficial activity in diabetic vascular dysfunction [21]. For the hypoglycemic effects, Rhodiola crenulata extract has been demonstrated to exert the glucose-lowering effect partly by inhibiting hepatic gluconeogenesis through activating the AMPK signaling pathway [37]. Furthermore, it has been demonstrated that salidroside possessed hypoglycemic activity and protective effects against diabetes-induced oxidative stress [12]. In addition, salidroside has been reported to regulate lipid metabolism in type 2 diabetic mice by down-regulation of miR-370 of primary hepatocytes [38], improve the cellular metabolic flux by the activation of a mitochondria-related AMPK/PI3K/Akt/GSK3β pathway in hepatocytes [39], regulate glucose homeostasis in obese mice by repressing inflammation in white adipose tissues and improving leptin sensitivity in hypothalamus [15], stimulate glucose uptake in skeletal muscle cells by activating AMPK, and alleviate diabetic albuminuria by downregulation of caveolin-1 phosphorylation and inhibition of albumin transcytosis across glomerular endothelial cells [19].

In this study, chronic administration of 100 mg/kg/day SAL not only significantly reduced glucose levels (Table 1), but also reduced blood pressure (Fig. 1) with a decreased contractile activity of cerebral artery (Figs. 2 and 3) in diabetic GK rats. In addition, chronic administration of 100 mg/kg/day SAL not only significantly decreased the whole-cell current densities of Ca_L_ channel (Fig. 4), but also reduced the expressions of Ca_L_ channel α_1C_-subunit at protein and mRNA levels (Fig. 5) in cerebral VSMCs of diabetic GK rats. Correspondingly, acute application of 100 μM SAL could directly induce cerebrovascular vasodilatation (Fig. 6) and decreased the Ca_L_ channel currents (Fig. 7) in cerebral VSMCs isolated from control WKY rats under hyperglycemia condition. Furthermore, SAL significantly inhibited the mRNA levels of Ca_L_ α_1C_-subunit and its downstream kinase, MLCK, in cultured cerebral VSMCs (Fig. 8). Our results clearly suggested SAL treatment alleviated cerebrovascular contractile activity in diabetic GK rats by inhibition of Ca_L_ channel in VSMCs.

It has been reported that daily oral gavages with SAL (40 mg/kg) for 5 weeks did not change the blood pressure in diabetic GK rats [21], which is similar to our observation that administration of 50 mg/kg/day SAL for 4 weeks did not affect the blood pressure in GK rats in *Experiment I*. However, we also found that dosage of 100 mg/kg/day SAL is effective for lowering blood pressure in diabetic GK rats and dosage of 200 mg/kg/day SAL lowered blood pressure not only in diabetic GK rats but also in control WKY rats (Fig. 1). Therefore, the dosage of SAL is very important for the treatment in diabetic GK rats.

### Practical implications of the present study

Several studies and new guidelines indicated that aggressive treatment of hypertension with 2 or more antihypertensive agents would reduce blood pressure and show a concomitant reduction of cardiovascular risk in diabetic subjects [5, 6]. For example, angiotensin-converting enzyme inhibitors, angiotensin-receptor blockers, thiazide diuretics, beta-adrenoreceptor blockers, and calcium-channel blockers, are effective antihypertensive agents in type 2 diabetes [6, 7]. Here, we observed for the first time that SAL, a traditional herb, could provide a combinational therapy for lowering blood glucose (Table 1) and reducing blood pressure (Fig. 1) in diabetic GK rats at the same time. Therefore, SAL treatment could be a good alternative or at least supplement to treat DM by lowering blood glucose and reducing cardiovascular risk at the same time in diabetic patients. In addition, it has been demonstrated that impaired Ca_L_ channel is associated with increased arterial contractility, which leads to the elevated blood pressure and reduced blood flow in diabetes [1, 22]. Our work found that SAL treatment alleviated cerebrovascular contractility by directly inhibiting Ca_L_ channel, which might be the underlying mechanism of SAL responsible for vascular protection in diabetes. Furthermore, inhibition of Ca_L_ channel may provide a novel target for therapeutic intervention for diabetic vascular complications in diabetes.

### Limitations of the study

Firstly, we did not investigate the concentration of salidroside in plasma after intragastric gavage (i.g) administration of 100 mg/kg salidroside in vivo in the present study. It is believed that β-glucosidases could hydrolyze the phenol glycoside to aglycone in the jejunum, and then aglycone are absorbed by the intestine or further metabolized into several other products before absorption by intestinal microflora [40]. It has been suggested that salidroside may metabolize to p-tyrosol, its sulfate or glucuronide conjugates, or even the methylate after i.g administration [41]. Furthermore, it has been reported that the absorption of salidroside in plasma reached the maximum within 20–30 min after i.g administration of 100 mg/kg salidroside and then decreased [41]. In animal study of present work, we administrated 100 mg/kg/day salidroside by i.g to GK and control rats for 4 weeks to investigate blood pressure and the function of Ca_L_ channel. In cell study of present study, we administrated 100 μM salidroside to cultured cerebral VSMCs for 48 h under hyperglycemia in vitro. The concentration of salidroside (100 μM) in our study is same to the previous report that 100 μM salidroside blocks the proliferation of pulmonary artery smooth muscle cells (PASMCs) induced by platelet-derived growth factor-BB in vitro [42]. In addition, the concentration of salidroside (100 μM) in our study is also similar to the previous report that 800 μM salidroside modulated cell apoptosis in mouse cultured pulmonary arterial smooth muscle cells (PASMCs) after chronic hypoxia exposure in vitro [10]. Secondly, Our results indicated that 100 μM SAL or 100 nM nifedipine induced the similar effects on mRNA expressions of Ca_L_ α1C-subunit in normal or high glucose condition (Fig. 8c). It has been reported that the sensitivities of Ca_L_ currents to nifedipine or Bay K 8644 were altered in tail artery SMCs from streptozotocin-induced diabetic rats [43]. Until now, only we investigated that salidroside could alter the function and expression of Ca_L_ channel in diabetes. In further work, it is very interesting to compare the effects of SAL and nifedipine on the expressions of Ca_L_ in GK and normal rats in vivo.

## Conclusion

There are two novel findings in the present work. First, chronic administration of 100 mg/kg/day SAL for 4 weeks significantly not only lowered blood pressure, but also reduced blood pressure and alleviated cerebrovascular contractile activity in diabetic GK rats, which suggested that SAL treatment might provide a combinational therapy for lowering blood glucose and reducing blood pressure in diabetes at the same time. Secondly, SAL treatment markedly inhibited the function and expression of Ca_L_ channel in cerebral VSMCs isolated from diabetic GK rats or when exposed to hyperglycemia condition, which may be the underlying mechanism responsible for the vascular protection of SAL in diabetes. Taken together, the present study provided evidences that SAL contributes to reducing blood pressure and alleviating cerebrovascular contractile activity in diabetic GK rats by inhibition of Ca_L_ channel in smooth muscle cells, which may provide a novel approach to treat vascular complications in diabetic patients.

## Additional files

Additional file 1: Fig S1. Comparison of systolic (**a**) and diastolic blood pressure (**b**) from WKY, WKY + SAL, GK, and GK + SAL rats. (XLSX 11 kb)

Additional file 2: Fig S2. Comparison of contractile function in response to 60 mM KCl (**a**) and 10^−6^ M 5-hydroxytryptamine (5-HT, **b**) in middle cerebral artery isolated from WKY, WKY + SAL, GK, and GK + SAL rats, respectively. (XLSX 11 kb)

Additional file 3: Fig S3. Chronic administration of 100 mg/kg/day SAL significantly inhibited the contractile responsiveness of middle cerebral artery to cumulative superfusion of KCl (**a**) and 5-hydroxytryptamine (5-HT, **b**) in diabetic GK rats. (XLSX 10 kb)

Additional file 4: Fig S4. Chronic administration of 100 mg/kg/day SAL markedly decreased whole-cell Ca_L_ current densities of cerebral VSMCs isolated from diabetic GK rats. (XLSX 9 kb)

Additional file 5: Fig S5. Chronic administration of 100 mg/kg/day SAL significantly reduced the expressions of Ca_L_ α_1C_-subunit at protein and mRNA levels in cerebral arteries isolated from diabetic GK rats. (XLSX 9 kb)

Additional file 6: Fig S6. Acute application of SAL directly induced vasodilatation of middle cerebral artery isolated from normal WKY rats by inhibition of Ca_L_ channel under hyperglycemia condition. (XLSX 8 kb)

Additional file 7: Fig S7. Acute application of SAL directly inhibited the Ca_L_ currents in cerebral VSMCs isolated from normal WKY rats under hyperglycemia condition. (XLSX 8 kb)

Additional file 8: Fig S8. Treatment with SAL for 48 h inhibited the mRNA expressions of Ca_L_ α_1C_-subunit and MLCK in cultured cerebral VSMCs under high glucose condition. (XLSX 9 kb)

## Abbreviations

[Ca^2+^]_i_: Concentration of intracellular Ca^2+^
5-HT: 5-hydroxytryptamine
Ca_L_: Long-lasting voltage-dependent Ca^2+^ (l-type Ca^2+^) channel
CICR: Ca^2+^-induced Ca^2+^ release
DM: Diabetes mellitus
SAL: Salidroside
VSMCs: Vascular smooth muscle cells
